# Why the Corvis Biomechanical Factor Should Only Be Used for Corneal Ectasia

**DOI:** 10.1167/tvst.12.5.24

**Published:** 2023-05-24

**Authors:** Robert Herber, Riccardo Vinciguerra, Elias Flockerzi, Paolo Vinciguerra, Berthold Seitz

**Affiliations:** 1 Department of Ophthalmology, Univ. Hospital Carl Gustav Carus, TU Dresden, Germany; 2 Humanitas San Pio X Hospital, Milan, Italy; 3 Department of Ophthalmology, Saarland University Medical Center, Homburg, Germany; 4 IRCCS Humanitas Research Hospital, Rozzano, Milan, Italy; 5 Humanitas University, Department of Biomedical Sciences, Pieve Emanuele, Milan, Italy. e-mail: robert.herber@ukdd.de

We read with great interest the study by Chou et al.^1^ entitled “Corvis Biomechanical Factor Facilitates the Detection of Primary Angle Closure Glaucoma.” The authors examined the Corvis Biomechanical Factor (CBiF) in a cohort of 79 healthy eyes and 81 eyes with primary angle closure glaucoma (PACG). It is known that corneal biomechanics are altered in primary open angle glaucoma and the need for such a study arises from its often asymptomatic course.^2^ Therefore, an application of these metrics might also be helpful for detecting PACG.

The main issue of the study design is the use of the CBiF as a measure of corneal biomechanical stability.^1^ The authors stated that the “CBiF reflects the overall biomechanical stability of the cornea in a linear manner,”^1^ which raises controversies that we would like to discuss in this letter.

First, the CBiF is derived from the Corvis Biomechanical Index (CBI), which was developed to detect keratoconus.^3^ Vinciguerra et al.^3^ used a dataset of healthy and keratoconus eyes to distinguish between these both cohorts. They used a logistic regression, whereby the dependent target variable “disease” was classified as “0” (=healthy) and “1” (=keratoconus) with a cutoff of 0.5. The included independent variables (features) were combined linearly using certain coefficients so that the result of the formula (CBI) separated between healthy and keratoconus eyes.^3^ The amount of the coefficients of each feature depends on the definition of the target variable. This approach is known as supervised machine learning.^4^ Therefore, the CBI is not a measure for overall corneal stability. Instead, it is created to separate between a normal or pathological condition. Obviously, the disease of keratoconus is strongly related to a biomechanical reduced or weakened cornea. Nevertheless, a general interpretation of a weaker or stiffer cornea should be avoided when using the CBI.

Subsequently, the CBiF, which was used in this study, is a linear version of the original CBI (CBI beta), which was previously defined by Flockerzi et al.^5^ Contrary to the author's statement,^1^ the CBiF does not reflect the overall corneal stability as it was designed to grade keratoconus severity. As a matter of fact, Flockerzi et al. investigated the relationship between the CBI beta and the anterior curvature, posterior curvature, and thinnest corneal thickness; they found higher values of CBI beta in steeper corneas (a smaller radius of curvature indicates a higher stage of keratoconus, a negative correlation). The strongest correlation was observed for posterior curvature, so the authors decided to match these two. Therefore, the CBiF is referred to the amount of the posterior corneal curvature.^5^

Third, when analyzing the dynamic behavior of the cornea in patients with glaucoma, it should be noted that it seems to be different from the behavior of keratoconus eyes. An indication for this is the pilot study of the Dresden Biomechanical Glaucoma Factor. In this study, most of the features (variables) used in the regression analysis were found to be different from the CBI formula, when applying a similar machine learning algorithm separating normal tension glaucoma from age-matched normal eyes.^3^^,^^6^ Moreover, Vinciguerra et al.^7^ showed a potential different dynamic response of the cornea in high tension glaucoma in comparison with normal tension glaucoma. As a result of this, the Dresden Biomechanical Glaucoma Factor or Biomechanical Glaucoma Factor should only be used for assessment in eyes with normal tension glaucoma.

We agree with the authors that corneal biomechanics are altered in PACG, but would like to invite them to present a statistical analysis of dynamic corneal response parameters of the Corvis ST such as deformation ratio (at 2 mm), integrated inverse radius, stiffness parameter at first applanation, and stress–strain index. These parameters better reflect the biomechanical properties of the cornea and might also differ between healthy eyes and eyes with PACG.

In summary, machine learning algorithms are novel tools that help physicians to detect diseases better and earlier. Especially in ophthalmology, these methods are flooding the market with the introduction of new diagnostic devices. It is necessary to be informed about those parameters and to know how they are developed and how they are working to avoid a misinterpretation in clinical practice, as well as in scientific purposes. Machine learning–based algorithms should be used for what they were trained for, leading to the conclusion that the CBiF should only be used for detecting and monitoring early ectasia and keratoconus.
